# Chronic neutrophilic leukemia with *JAK2* mutation: is it true chronic neutrophilic leukemia?

**DOI:** 10.1007/s00277-023-05376-2

**Published:** 2023-07-31

**Authors:** Jiapei Gao, Junyin Gao, Fengling Min

**Affiliations:** 1 Department of Hematology, The Affiliated Hospital of Yangzhou University, Yangzhou University, Yangzhou, Jiangsu Province China; 2 Pulmonary and Critical Care Medicine, Yancheng No. 1 People’s Hospital, Yancheng, Jiangsu Province China; 3 Department of Hematology, Yangzhou Hospital of Traditional Chinese Medicine, Yangzhou, Jiangsu Province China

Dear Editor,

Recently, we read an interesting report in this journal in which a chronic neutrophil leukemia (CNL) patient carried *JAK2* mutation [1]. Almost all patients with polycythemia vera (PV) carry *JAK2* mutations, and *JAK2* V617F also occurs in essential thrombocythemia (ET) and primary myelofibrosis (PMF), with mutation frequencies of 55% and 65%, respectively [2]. Although oncogenic driver mutations in *CSF3R* remain the genetic signature of CNL, approximately 10% of *CSF3R* mutations in CNL patients are negative [3]. By searching databases, a total of 12 CNL patients carrying *JAK2* mutations were found (Supplemental material). The median hemoglobin level was 135 g/L, which was higher than the majority of CNL patients [3], and their clinical phenotype was similar to that of PV; there may be three possible reasons accounting for this phenomenon.

First, different orders of mutation acquisition may cause different clinical phenotypes. In recent years, with the development of next-generation sequencing (NGS) technology, a large number of non-driver gene mutations related to prognosis and therapy have been discovered, and the order in which they are acquired may affect the clinical phenotypes and therapeutic effects of myeloproliferative neoplasms (MPN) [4]. The order of *DNMT3A* acquisition may affect the phenotype of MPN. When it occurs before *JAK2*, it often shows the ET phenotype, and when it occurs after, it is more inclined to the PV phenotype [5]. In addition, when *TET2* occurs before *JAK2*, it will lead to the ET phenotype, and when the *JAK2* mutation is acquired first, it tends to have a more PV phenotype, the risk of thrombosis is increased, and *JAK2*-mut progenitor in vitro sensitivity to ruxolitinib is also increased. Ortmann believes [6] that the initial mutation may modify the epigenetic program of hematopoietic stem cells and progenitor cells and thus alter the consequences of the second mutation.

Second, these *JAK2*-mut CNLs may be the “intermediate form” in the evolution of PV to CNL. Approximately 5% of ETs can evolve into PV, PV can evolve into myelofibrosis, and PV can also directly transform into acute myeloid leukemia (AML) [7]. Merchant believes that *JAK2* V617F-mut ET, PV, and PMF form a biological continuum. In this continuum, ET and PV are in the chronic phase of MPN, PMF is in the accelerated phase of MPN, and secondary AML is in the acute phase of MPN [7]. In early years, Higuchi summarized 6 cases of PV that evolved into CNL [8]; the median time to evolve into CNL was 9.5 years. When evolved into CNL, hemoglobin and platelets were significantly decreased, while leukocytes were significantly increased. Due to the detection technology at that time, *JAK2* V617F was not detected in any patients. In the ensuing 15 years, no similar cases were reported until Castelli reported a PV patient with CNL phenotype after 5 years of hydroxyurea treatment in 2014 [9]. Although this patient was negative for *CSF3R*, their current examination supported the diagnosis of CNL. Castelli believes that it is not true CNL but an uncommon evolution of PV. The clinical manifestations of persistent mature neutrophilia and thrombocytosis may be consistent with the early phase of myelofibrosis, as well as the evolution to CNL.

Third, these *JAK2*-mut CNLs may have been misdiagnosed in the past. Barbui et al. found that among the patients initially diagnosed with *JAK2*-mut ET, a small number of people had phenotypic changes during the course of the disease and progressed to PV. Such patients were called masked PV (mPV). Compared with PV patients, these mPV patients had lower hemoglobin levels, and mPV patients were actually in the early phase of PV and misdiagnosed as *JAK2*-mut ET [10]. Therefore, in 2016, the WHO revised the diagnostic criteria for PV and lowered the hemoglobin threshold to 165 g/L (male) and 160 g/L (female) [11].

In short, with a deeper understanding of molecular biology and clonal evolution in MPN, the WHO will refine the diagnosis and classification of CNL in the future. We need to carefully identify subtypes of MPN in clinical work, especially *JAK2*-mut MPN with “CNL” clinical manifestations.

### Supplementary Information

Below is the link to the electronic supplementary material.

Supplementary file1 (DOCX 30 KB)

## Data Availability

The authors confirm that the data supporting the findings of this study are available within supplementary materials.
