# Supplementary material for: Chronic neutrophilic leukemia with JAK2 mutation: is it true chronic neutrophilic leukemia?
Source: Ann Hematol. 2023 Jul 31;102(11):3275–6. doi: 10.1007/s00277-023-05376-2 (PMC10567908; doi:10.1007/s00277-023-05376-2)
Supplement: Supplementary file 1 — Supplementary file1 (DOCX 30 KB) [file 277_2023_5376_MOESM1_ESM.docx]

By searching English databases PubMed, Web of Science, Medline, Springer-Link, Wiley, Google Scholar and Chinese databases China National Knowledge Infrastructure (CNKI), China Biology Medicine disc (CBMdisc), Wanfang Database, China Science and Technology Journal Database (VIP), Baidu Scholar, using "*JAK2* V617F" and "chronic neutrophilic leukemia" as keywords, found 9 relevant articles from 2015 to 2023, and a total of 12 cases of CNL patients carrying JAK2 mutations, of which references 1, 2, and 5 are in Chinese, the rest are in English.

**Laboratory features of 33 cases with *JAK2*-mut CNL patients**

| **Published years, study and reference** | **Age(year)/Sex** | **WBC (×10^9^/L)** | **HB (g/L)** | **PLT (×10^9^/L)** | **Clinical features and treatment** | **Status and Survival (months)** |
| --- | --- | --- | --- | --- | --- | --- |
| 2015，Lu et al. [1] | - | 29.8 | 135 | 335 | splenomegaly, *CSF3R*,  *ASXL1* (+), treated with Hu | Alive; 5 |
| 2015，Li et al. [2] | 69/M | 30.4 | 92 | 119 | bloating, weight loss, hepatosplenomegaly, *CSF3R* (-), treated with Hu | - |
| 2016，Hu et al. [3] | 64/M | 25.2 | 191 | 289 | fatigue, weight loss, splenomegaly, treated with Hu | Alive; 24 |
| 2018，Mendiola et al. [4] | 61/M | 36.2 | 82 | 117 | night sweats, weight loss, hepatosplenomegaly, *SRSF2* (+), died due to severe infection | Dead; 1 |
| 2018，Yu et al. [5] | 52/M | 45 | 152 | 124 | night sweats, splenomegaly, *CSF3R*, *CALR*, *MPL* (-), treated with Hu and IFN | - |
| 2019，Yin et al. [6] | 53/F | 29 | 142 | 38 | splenomegaly, *CSF3R*, *ASXL1*, *SETBP1*, *CALR* (-) | Alive; 49 |
|  | 79/F | 61.7 | 130 | 211 |  | Alive; 59 |
| 2019，Zhang HJ et al. [7] | - | - | - | - | *ASXL1*, *EZH2*（+） | - |
|  |  |  |  |  | *ASXL1*, *PPM1D*, *PHF6*（+） |  |
|  |  |  |  |  | *SRSF2*, *TET2*（+） |  |
| 2020，Kakkar et al. [8] | 70/F | 24.7 | 155 | 129 | gout, fever, hepatosplenomegaly, treated with Hu | Alive; 18 |
| 2022，Lauw et al. [9] | 88/F | 52.7 | 86 | 205 | *ASXL1* (+), associated with MGUS, died due to HCC | Dead; 1 |

**Abbreviation**: WBC: white blood cell; HGB: hemoglobin; PLT: platelet; Hu: hydroxyurea; IFN: interferon; MGUS: monoclonal gammopathy of undetermined significance;

HCC: hepatocellular carcinoma

**References**

1. Lu B, Liu ZL, Zhou HH, Jin MD (2015) Analysis on the Clinical Features and CSF3R Mutations of Chronic Neutrophilic Leukemia. Anti-Tumor Pharmacy (3):185-188. doi:10.3969/j.issn.2095-1264.2015.037

2. LI Q, Fan L, Gao BH (2015) A case of chronic neutrophilic leukemia with JAK2 V617F mutation. Yinanbing Zazhi (5):533-533. doi:10.3969/j.issn.1671-6450.2015.05.029

3. Hu J, Xu Y, Li Z, Hong X, Lu Q (2016) Chronic neutrophilic leukemia with JAK2 V617F mutation: A case report. Int J Clin Exp Pathol 9(7): 7482-7487.

4. Mendiola VL, Qian YW, Jana B (2018) Septic Shock Predisposed by an Underlying Chronic Neutrophilic Leukemia with an Atypical Presentation; A Case Report. Case Rep Oncol 11 (3):871-879. doi:10.1159/000495458

5. Yu XB, Chen LY, Wang LJ, Wang W (2018) A case of chronic neutrophilic leukemia with JAK2 V617F mutation. Journal of Harbin Medical Unversity 52 (04):402-403

6. Yin B, Chen X, Gao F, Li J, Wang HW (2019) Analysis of gene mutation characteristics in patients with chronic neutrophilic leukaemia. Hematology 24 (1):538-543. doi:10.1080/16078454.2019.1642554

7. Zhang H, Wilmot B, Bottomly D, Dao KT, Stevens E, Eide CA, Khanna V, Rofelty A, Savage S, Reister Schultz A, Long N, White L, Carlos A, Henson R, Lin C, Searles R, Collins RH, DeAngelo DJ, Deininger MW, Dunn T, Hein T, Luskin MR, Medeiros BC, Oh ST, Pollyea DA, Steensma DP, Stone RM, Druker BJ, McWeeney SK, Maxson JE, Gotlib JR, Tyner JW (2019) Genomic landscape of neutrophilic leukemias of ambiguous diagnosis. Blood 134 (11):867-879. doi:10.1182/blood.2019000611

8. Sugandha, Kakkar N, Joseph John M (2020) Chronic neutrophilic leukemia presenting as secondary gout: Report of a rare myeloproliferative disorder. Indian J Cancer 57 (2):201-204. doi:10.4103/ijc.IJC_560_18

9. Lauw MIS, Hakim N, Arora S, Prakash S, Xie Y (2022) A case of plasma cell neoplasm-associated chronic neutrophilic leukemia with ASXL1 and JAK2V617F mutations. Ann Hematol 101 (8):1879-1881. doi:10.1007/s00277-022-04834-7
